# Thermal Plasticity and Evolutionary Constraints in *Bacillus*: Implications for Climate Change Adaptation

**DOI:** 10.3390/biology13121088

**Published:** 2024-12-23

**Authors:** Enrique Hurtado-Bautista, Africa Islas-Robles, Gabriel Moreno-Hagelsieb, Gabriela Olmedo-Alvarez

**Affiliations:** 1 Departamento de Ingeniería Genética, Unidad Irapuato, Cinvestav 36824, Mexico; enrique.hurtado@cinvestav.mx (E.H.-B.); africa.islas@cinvestav.mx (A.I.-R.); 2 Department of Biology, Wilfrid Laurier University, Waterloo, ON N2L 3C5, Canada; gmoreno@wlu.ca

**Keywords:** experimental evolution, critical high temperature, phenotypic plasticity, norms of reaction to temperature, convergent evolution, c-di-AMP, upper thermal limit, evolutionary rescue, thermal plasticity, thermal niche

## Abstract

The ongoing rise in global temperatures poses significant challenges to ecosystems, particularly impacting bacterial communities that are central to biogeochemical cycles. The resilience of wild mesophilic bacteria to temperature increases of 2–4 °C remains poorly understood. In this study, we conducted experimental evolution on six wild *Bacillus* strains from two lineages (*Bacillus cereus* and *Bacillus subtilis*) to examine their thermal adaptation strategies. We exposed the bacteria to gradually increasing temperatures to assess their thermal plasticity, focusing on the genetic mechanisms underlying adaptation. While *B. subtilis* lineages improved growth at highly critical temperatures, only one increased its thermal niche to 4 °C above their natural range. This finding is concerning given climate change projections. *B. cereus* strains exhibited higher mutation rates but were not able to grow at increasing temperatures, while *B. subtilis* required fewer genetic changes to increase heat tolerance, indicating distinct adaptive strategies. We observed convergent evolution in five evolved lines, with mutations in genes involved in c-di-AMP synthesis, which is crucial for potassium transport, implicating this chemical messenger for the first time in heat tolerance. These insights highlight the vulnerability of bacteria to climate change and underscore the importance of genetic background in shaping thermal adaptation.

## Simple Summary

As global temperatures rise due to climate change, understanding how bacteria respond to heat is essential. Bacteria are critical to ecosystems, helping recycle nutrients, break down organic matter, and support plant growth. If they struggle to adapt to warmer conditions, the effects could ripple through ecosystems. Our study tested whether *Bacillus* bacteria, common in soil and water, could adapt to gradual temperature increases. We exposed seven strains from two groups to experimental evolution under increasing warming conditions, expecting them to evolve heat tolerance. Although some bacteria showed small improvements in heat tolerance, neither group could evolve to grow well at temperatures just three degrees Celsius above their natural range. This is concerning, as climate change could raise global temperatures by 2–4 °C, which could push these bacteria beyond their limits. We also found that c-di-AMP, a molecule that helps regulate potassium levels in cells, was involved in temperature tolerance. These findings highlight the vulnerability of essential bacteria to climate change and underscore the need for further research to predict and mitigate its impacts on ecosystems.

## 1. Introduction

Bacteria are often regarded as highly adaptable to extreme environmental changes, including rising temperatures driven by global warming. However, this perception oversimplifies the complexity of bacterial adaptation and does not account for the millions of years it has taken for the diversity of bacteria to adapt to particular niches. This highlights that their adaptive capacity is deeply rooted in long evolutionary processes.

Understanding bacterial responses to heat stress is critical, given their central role in nutrient cycling and ecosystem stability, especially as global temperatures are projected to increase by 2 to 4 °C [1,2]. The impact of rising temperatures extends beyond bacterial physiology, with significant consequences for ecosystems and biogeochemical cycles; for example, elevated ocean temperatures alter nitrogen-fixing microbial populations, threatening the nitrogen cycle in coral reefs [3]. Similarly, warming freshwater ecosystems decreases bacterial diversity, disrupting ecological balance [2]. Rising temperatures are also linked to the spread of antibiotic resistance [4,5] and increased abundance of pathogens like *Vibrio cholerae* in coastal waters [6]. These examples underscore the urgency of understanding bacterial thermal adaptation in the context of global climate change.

Phenotypic plasticity—the ability of organisms to adjust their traits in response to changing environmental conditions [7,8]—allows species to buffer against environmental fluctuations. This adaptability can extend an organism’s ecological range, potentially leading to genetic assimilation, where initially flexible traits become genetically stabilized [9]. Nevertheless, plasticity has inherent limitations and costs, especially under sustained stress [10]. Reaction norms, which describe how a genotype’s phenotype changes across environmental gradients, are valuable tools for assessing thermal limits and understanding adaptive strategies. We can learn about a given organism’s thermal niche through reaction norms (Wolterek, 1909 [11], cited by Stearns [12]). Mesophilic bacteria, such as *Escherichia coli* [13] and some *Bacillus* species [14], typically thrive between 27 °C and 40 °C.

When exposed to higher temperatures, mesophilic bacteria activate a heat-shock response (HSR), which involves the rapid production of heat-shock proteins that stabilize cellular components and temporarily protect against heat stress [15,16]. However, these adaptations are generally short-lived and insufficient for prolonged survival under extreme temperatures. *Escherichia coli*, as a well-established model organism, has been extensively studied for its physiological responses to thermal stress. Unlike thermal tolerance, which temporarily protects cells from heat damage by activating heat-shock proteins [17], thermal adaptation requires genetic changes that can withstand and sustain growth at higher temperatures beyond the initial threshold [18].

Experimental evolution is a powerful tool for gaining insights into the capacity of bacteria to adapt to specific biotic and abiotic challenges and understanding the types of mutations that shape or enable such adaptations to changing environments [18,19]. It allows researchers to address a key question in evolutionary biology: whether bacteria can evolve similar phenotypic outcomes through the same or different genetic mechanisms—a phenomenon known as convergent evolution [20,21]. Convergent evolution can manifest in multiple ways, including traits that arise via a conserved genetic mechanism within a clade or through distinct mechanistic bases even among closely related species.

Previous experiments have documented a rapid adaptive response of *E. coli* to high temperatures [22], with evidence that the genetic response can be diverse [23]. Similarly, Tenaillon et al. [24] took a whole-genome approach to estimate the extent of evolutionary convergence that occurs during adaptation in 115 populations of *E. coli* at a high temperature of 42.2 °C, analyzing the overall genetic basis of adaptation. Their findings highlighted convergence among genes, with the most mutated gene being *rpoB*, along with proteins that regulate the *rpoS* stress response (RSS).

Although numerous studies have explored the ability of *E. coli* to evolve at different temperatures [25], few studies have aimed to evolve bacteria to expand their thermal niche. In this context, Murata et al. [26] subjected an *E. coli* strain to 43 °C and identified 51 genes crucial for thermotolerance, highlighting the diverse genetic pathways involved in developing heat tolerance. Murata introduced the term “critically high temperatures” (CHT) to describe temperatures beyond the thermal limit.

Bennett and Lenski [13] sought to isolate evolved lines capable of surpassing the upper thermal limit and found that this limit did not significantly increase in any of the derived lines, even those that evolved at 41–42 °C. Exposure to 44 °C proved lethal, except for one thermotolerant mutant, which managed to grow at 44 °C [27]. Mongold et al. [28] further investigated evolutionary adaptation to what they termed a “lethal thermal environment” by directly shifting evolution to 44 °C. Their findings revealed that three thermotolerant mutants were derived from two progenitor lines that had previously adapted to 41–42 °C, suggesting that these lines extended their upper thermal limit in a stepwise manner relative to their progenitors. Additionally, they noted that not all evolved lines exhibited trade-offs, such as a loss of fitness at lower temperatures.

*Bacillus* strains are particularly important to study in the context of prolonged survival at temperatures exceeding their niche range because they are ubiquitous and ecologically significant, playing key roles in decomposing organic matter and nutrient cycling. Although *Bacillus* can form heat-tolerant spores that remain dormant for decades, their heterotrophic functions occur only in a vegetative state. Therefore, understanding whether *Bacillus* can evolve more heat-tolerant vegetative cells is crucial for predicting how these bacteria will respond to global warming. Recent research has shown that wild *Bacillus* strains exhibit considerable variation in thermal tolerance, influenced by genetic background [14], emphasizing that temperature is a critical factor affecting bacterial growth and survival. Despite extensive research on acute bacterial responses, the long-term evolution of bacterial thermotolerance and the upper thermal limits they can achieve remain poorly understood and research has not been conducted on *Bacillus* lineages.

This study addresses this knowledge gap by investigating whether wild *Bacillus* strains from two distinct lineages can evolve heat tolerance in their vegetative cells through experimental evolution. We aim to determine whether gradual temperature increases enable these bacteria to develop the plasticity needed to survive projected global temperature rises of 2 to 4 °C and to identify the genetic mechanisms underlying their adaptations. By focusing on different lineages and genetic backgrounds, our research explores the constraints and potential pathways for bacterial adaptation to extreme heat, providing insights into their evolutionary resilience and broader ecosystem impacts.

The main conclusions of this work are as follows: 1. The observed lineage-specific outcomes and convergence within strains underscore the critical role of genetic background in shaping adaptive responses. *B. cereus* and *B. subtilis* have different plasticity and *B. cereus* lineages, contrary to those of *B. subtilis*, and were not able to increase thermal tolerance relative to ancestral lines. Only one evolved *B. subtilis* line successfully extended its thermal niche. 2. Tolerance to higher temperatures was observed in *B. subtilis* lines that evolved constantly at 37 °C, indicating a metabolic adjustment driven by continuous growth conditions and the growth medium rather than selective pressure from increased temperatures. 3. Convergent mutations in genes regulating c-di-AMP synthesis uncover, for the first time, its critical role in thermal tolerance. This discovery links molecular mechanisms to thermal adaptation across lineages. 4. Finally, our findings warn about bacteria’s inability to evolve significant thermal tolerance, even under gradual selection. This raises concerns about bacterial communities’ resilience to climate change and the potential cascading effects on biogeochemical cycles and ecosystem stability.

## 2. Materials and Methods

### 2.1. Strains and Microbial Methods

*Bacillus* strains were originally isolated from Cuatro Cienegas, Coahuila, México, as described [29]. The strains were collected from sediment in the Churince water system. Three strains grouped with the *B. cereus lineage* were 99% similar based on sequence variation of the 16S rRNA gene (accession numbers Bc_370a MK850162.1, Bc_102 MK880370, and Bc_111b MK850167.1), as well as four strains from the *B. subtilis* lineage (accession numbers Bs_21 MK850173.1, Bs_90 MK850153.1, and Bs_427 MK850159.1 and laboratory strain PY79 MK850170.1). Accession numbers for the genomes of the strains are:

SAMN09761860: *B.subtilis*_21_anc_ehb001 (TaxID: 1423);
SAMN09761861: *B.subtilis*_90_anc_ehb002 (TaxID: 1423);
SAMN09761862: *B.subtilis*_427_anc_ehb003 (TaxID: 1423);
SAMN09761863: *B.subtilis*_PY79_anc_ehb004 (TaxID: 1423);
SAMN09761864: *B.cereus*_370a_anc_ehb005 (TaxID: 1396);
SAMN09761865: *B.cereus*_102_anc_ehb006 (TaxID: 1396);
SAMN09761866: *B.cereus*_111b_anc_ehb007 (TaxID: 1396).

Strains were preserved at −70 °C in a marine medium [30] supplemented with 80% glycerol. For experimental use, strains were revived by streaking onto marine medium agar plates (1.6% agar) and incubated overnight at 28 °C. Single colonies were transferred into a liquid marine medium and incubated at 37 °C overnight to establish starting cultures.

### 2.2. Plate Incubation Experiments

Temperature tolerance was assessed by streaking the strains onto marine medium agar plates and incubating them at 37 °C, 44 °C, and 55 °C for 24 h. After the initial incubation, plates were transferred to 28 °C and incubated for an additional 48 h. Photographs were taken at both incubation stages to document growth and colony morphology.

### 2.3. Experimental Evolution

Seven *Bacillus* strains with previously characterized thermal tolerance [14] were selected, comprising four strains from the *B. subtilis* lineage (three wild isolates and the lab strain *B. subtilis* PY79) and three strains from the *B. cereus* lineage. Ancestral strains were frozen as reference samples. Experimental evolution was conducted in 50 mL Falcon tubes with cultures agitated at 150 rpm. For each strain, three populations evolved at a constant temperature of 37 °C, while another three populations were exposed to an incremental temperature regime starting at 43 °C and increasing to 47 °C over a period of 90 days.

**Figure 1:**
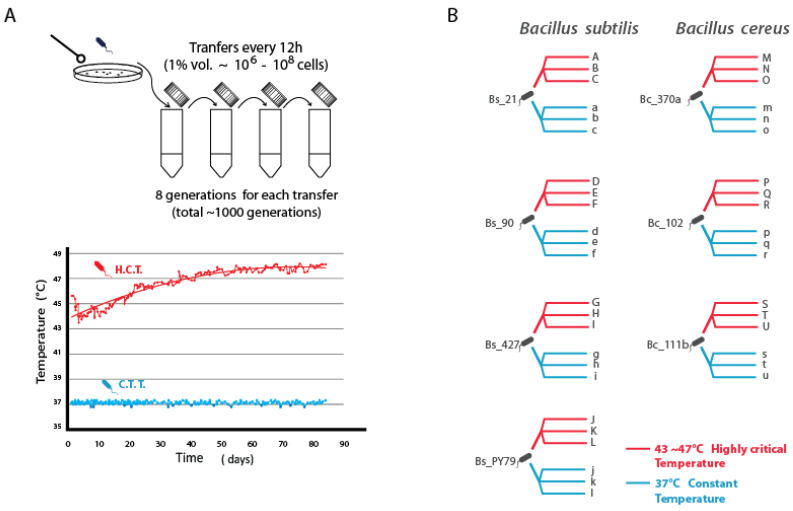
Experimental setup and lineage descent. (**A**) Inoculation and transfers were performed every 12 h until reaching approximately 1000 generations (eight generations per transfer). The plot illustrates the measured temperature profiles of the incubator over 1000 generations both for the population evolved under highly critical temperature (HCT) and the constant (lower) temperature treatment (CTT) at 37 °C. The mathematical model for HCT is represented as 4.04 + 0.59(t^0.5^), with an R^2^ value of 0.8685. (**B**) The figure depicts the four parental strains from the *B. subtilis* lineage (left) and their respective descending lines labeled from A to L (HCT) and a to l (CTT_37 °C). Similarly, three parental strains from the *B. cereus* lineage (right) are depicted along with their corresponding descending lines named from A to L (HCT) and a to l (CTT_37 °C).

Cultures were diluted 100-fold [31] every 12 h by transferring 50 µL of the culture into 5 mL of fresh marine medium, achieving approximately 1000 generations over 105 days. Generation counts were estimated based on viable cell numbers at saturation and calculated division rates. Turbidity measurements were taken every 12 h. In the incremental temperature treatment, the temperature was raised by 0.1 °C per day, contingent on observable growth (turbidity). If growth rates decreased significantly, indicated by low turbidity, temperature increases were paused or reduced, and inoculum volume was adjusted from 50 µL to 100 µL as needed. Temperature adjustments over time were recorded and represented as a parabolic curve (Figure 1A). Weekly samples of each evolving population were stored at −70 °C in 80% glycerol. At the experiment’s conclusion, colonies from each of the 42 evolved populations were isolated, and a representative evolved strain from each population was selected for further analysis.

### 2.4. Norms of Reaction to Temperature

Reaction norms for parental and evolved strains were determined using growth kinetics at various temperatures. Doubling times were calculated from the exponential growth phase to plot the growth rate against temperature. Strains were reactivated from −70 °C stocks on marine medium agar and incubated at 37 °C for 20 h. A single colony was used to inoculate 5 mL of marine medium broth and incubated overnight at 37 °C with shaking. Cultures were transferred to achieve exponential phase by subculturing 50 µL into fresh broth for 2 h.

Growth measurements were performed using a Bioscreen C plate reader (Labsystems, Helsinki, Finland) with a 420–580 nm filter. Each well of a 200-well microtiter plate was inoculated with 5 µL of culture and 175 µL of fresh marine medium broth, with three technical replicates per condition. Optical density was recorded every 30 min for 20 h. Growth kinetics were assessed at 17 °C, 27 °C, 37 °C, 41 °C, 43 °C, 46 °C, 49 °C, and 55 °C. Doubling times were calculated using exponential growth models. Reaction norms were compared using *t*-tests and multiple ANOVA tests (significance level 0.05) in Statgraphics 15.2.06 and R version 3.6.2.

### 2.5. Genome Sequencing and Data Analysis

DNA was extracted using the QuickGene DNA tissue kit S (Kurabo Industries LTD., FujiFilm, Osaka, Japan). Sequencing was conducted at Cinvestav-Langebio on an Illumina MiSeq platform at 50× coverage in a paired-end format, with ~180 bp fragment sizes. Paired-end reads were quality-checked using FastQC and trimmed with Trimmomatic. Genome assemblies were generated with SPAdes v3.13.0 and evaluated for quality using N50 and %GC content.

Variant calling was performed using GATK Haplotype Caller, and mutations were annotated with Prokka and Patric. Custom scripts were used to integrate Prokka GFF files with VCF data from GATK. Additionally, we employed a “fragmentome” analysis to ensure correct linkage of evolved genomes to their respective parental strains. In this approach, we first generated sets of diagnostic DNA fragments (1000 bp each) from the original ancestral genome sequences. By comparing these fragments against all assembled genomes, we identified those that were uniquely present (i.e., matched only their strain of origin) and thus served as reliable strain-specific genetic markers. Using these diagnostic fragments, we could confidently map evolved reads back to the appropriate parental genome. Unlike standard alignments to a single reference genome, this method provides a lineage-specific signature that helps distinguish closely related ancestral strains and their evolved descendants. This ensures that any potential cross-contamination or mislabeling during experimental evolution or sequencing could be easily detected, as evolved strains must match their diagnostic fragments and not produce hits against other parental genomes. Assembled reference genomes of each representative ancestral strain were obtained, making it possible to generate these strain-specific diagnostic fragments prior to the mapping of evolved sequences.

Variants were filtered with a quality threshold of 100, and low-quality mutations (<40) were excluded. Mutation categories included intergenic, genic, indels, and single substitutions. Mutations of interest were validated by Sanger sequencing. Annotated mutations were analyzed using UniProt, GO, and PFAM databases. Gene lists, oligonucleotide sequences, and accession numbers for sequencing data for evolved strains are provided in Supplementary Tables.

### 2.6. Sporulation Capacity

Sporulation was assessed on marine medium plates after seven days at room temperature. Samples were stained with malachite green and examined under a Leica 020-518.500 DM/LS optical microscope (Wetzlar, Germany) to confirm the presence or absence of spores.

## 3. Results

### 3.1. Experimental Evolution at Highly Critical Temperatures and Evolutionary Rescue

We conducted experimental evolution to test if the thermal tolerance of vegetative *Bacillus* strains could be enhanced through gradual temperature increases (Figure 1A). Our study included seven strains from two lineages: three *Bacillus cereus* strains and four *Bacillus subtilis* strains, both of which are part of strongly supported monophyletic clades within the *Bacillaceae* family [32,33]. The wild strains were originally isolated from a temperate hydrological system [29,34], and previous research indicated that these strains are mesophilic, with thermal tolerance largely influenced by lineage [14].

We hypothesized that a gradual increase in temperature would serve as a selective force, potentially expanding the thermal tolerance limits of these strains. With genetic variation among the seven strains, we anticipated diverse adaptive evolutionary strategies. The experimental design involved two temperature regimes: one with a constant temperature of 37 °C and another with incremental increases from 43 °C, aiming for approximately 1000 generations (Figure 1B).

Growth at 43 °C initially varied significantly among strains. Specifically, *B. cereus* strains were highly sensitive, unable to sustain growth beyond 48 h at this temperature, while *B. subtilis* strains exhibited greater resilience. For *B. cereus*, growth restoration occurred after lowering the temperature to 42 °C, suggesting an evolutionary rescue mechanism. *B. subtilis*, in contrast, maintained growth more consistently, allowing for temperature increments of 0.1 °C per day.

Temperature increments continued until the strains reached a plateau at 45 °C after approximately 300 generations and then slowed down (Figure 1A). The increment frequency decreased further until no additional adaptation was observed over the last 300 generations, indicating a stabilization of phenotypes. *B. subtilis* lineages showed a greater overall capacity to survive and adapt than *B. cereus*, consistent with their initial phenotypic plasticity.

### 3.2. Differences in Plasticity and Critical Temperature Limits

To assess changes in thermal tolerance, we compared the reaction norms of evolved and parental strains. Growth kinetics were measured across a temperature range from 17 °C to 55 °C and doubling times were used to construct growth rate curves.

#### 3.2.1. Plasticity and Critical Temperature Limits for *Bacillus subtilis* Lineages

**Figure 2:**
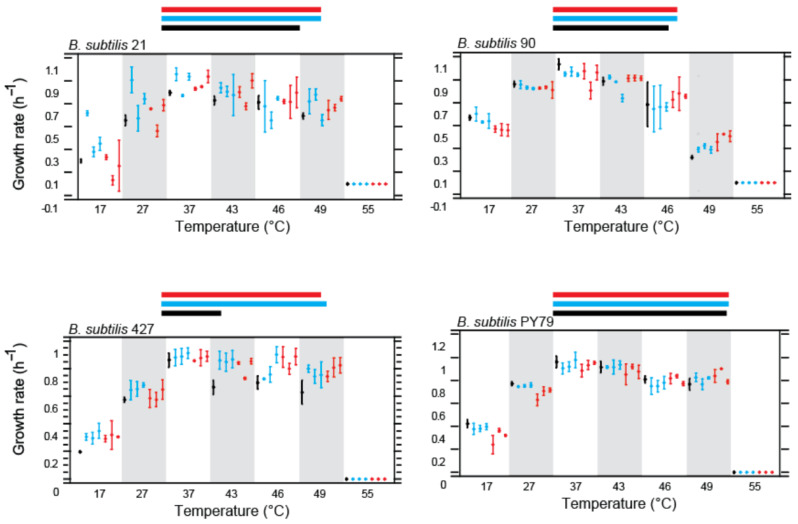
Norms of reaction *Bacillus subtilis* parental strains (black) and their respective evolved lines at HCT treatment (red) and CTT treatment (37 °C) (blue). Vertical bars are confidence intervals; qt 95% (ANOVA analysis, *p* = 0.05). Horizontal lines at the top of each graph represent the temperature niche of the ancestral strains (black) and strains evolved under CTT (blue) or HCT treatments (red).

**Figure 3:**
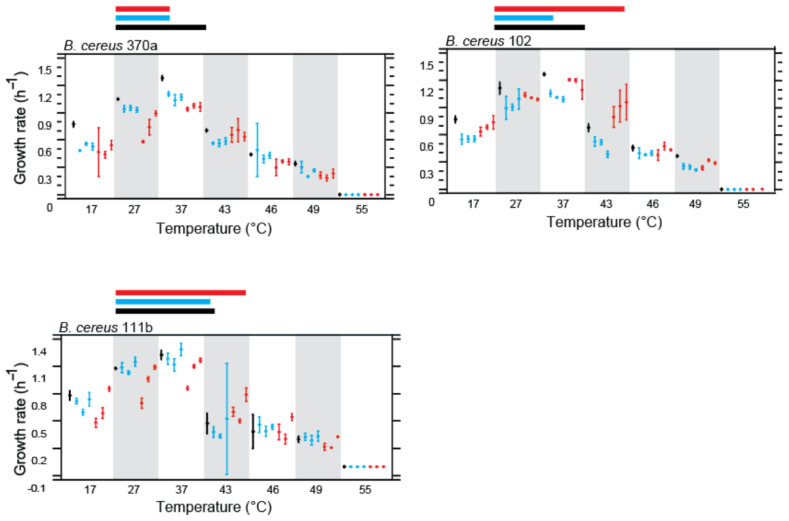
Norms of reaction *Bacillus cereus* parental strains (black) and their respective evolved lines upon HCT treatment (red) and CTT treatment (37 °C) (blue). Vertical bars are confidence intervals; qt 95% (ANOVA analysis, *p* = 0.05). Horizontal lines at the top of each graph represent the temperature niche of the ancestral strains (black) and strains evolved under CTT (blue) or HCT treatments (red).

Figure 2 and Figure 3 illustrate the reaction norms for *B. subtilis* strains. The Bs_21 and Bs_427 strains demonstrated the most substantial increases in tolerance at higher temperatures. Notably, several Bs_21 lines grew successfully at up to 49 °C, although the results varied among lines (a-b-c-A-C). All Bs_427-evolved lines showed improved growth at 46 °C and 49 °C, regardless of whether they were evolved under constant temperature treatment (CTT) or the high critical temperature (HCT) regime. This suggests that adaptation may be influenced by factors related to the growth medium. A statistically significant reduction in growth was observed for some Bs_427 lines (B and b) at 37 °C.

Bs_90 exhibited limited improvement in thermal tolerance, with only minor increases in growth at 43 °C for the D-E-d-e lines and barely significant growth at 46 °C for the D-E-F lines. The Bs_90 strain was the least capable of growing at 49 °C, though all evolved lines still outperformed the parental strain. A trade-off was evident in Bs_90, with all evolved lines showing reduced growth at lower temperatures (17–37 °C). In contrast, the laboratory strain Bs_PY79 showed consistent growth across temperatures up to 37 °C. While some high-temperature-evolved lines (J-K-L) demonstrated improved tolerance at 49 °C, they experienced a marked reduction in growth at 17 °C and 27 °C. Interestingly, all Bs_PY79 lines performed similarly to the parental strain at 43 °C, without surpassing its tolerance at 46 °C.

#### 3.2.2. Plasticity and Critical Temperature Limits for *Bacillus cereus* Lineages

For *B. cereus*, the reaction norms revealed minimal improvements post-evolution. Strain Bc_370a exhibited poorer growth than the parental strain, particularly for lines evolved under the HCT regime, which showed decreased growth at 27 °C and 37 °C. Bc_102 demonstrated the best improvements in thermal tolerance among HCT-evolved lines, though CTT-evolved lines generally showed reduced growth compared to the parental strain. Bc_111b presented a slight increase in growth at 43–46 °C for line U, indicating potential thermal adaptation. However, the S-T-U lines (HCT-evolved) exhibited a reduction in fitness across a lower temperature range (17–39 °C), suggesting maladaptive consequences of high-temperature evolution.

### 3.3. Changes in Carrying Capacity (K) and Growth Rate (G)

To further understand the adaptive strategies of evolved *Bacillus* strains, we assessed changes in two critical growth parameters: carrying capacity (K) and specific growth rate (G) at 37 °C and 43 °C. These measurements provided insights into whether the adaptations were multifactorial, impacting not just the growth rate but also the overall population density the cultures could sustain. The Principal Component Analysis (PCA) of experimental evolution treatments of the traits evaluated for each strain at both 37 °C and 43 °C highlighted the phenotypic responses and adaptations under different temperature regimes (Supplementary Figure S4).

#### 3.3.1. Impact of Temperature on Growth and Carrying Capacity on *Bacillus subtilis* Lineages

**Figure 4:**
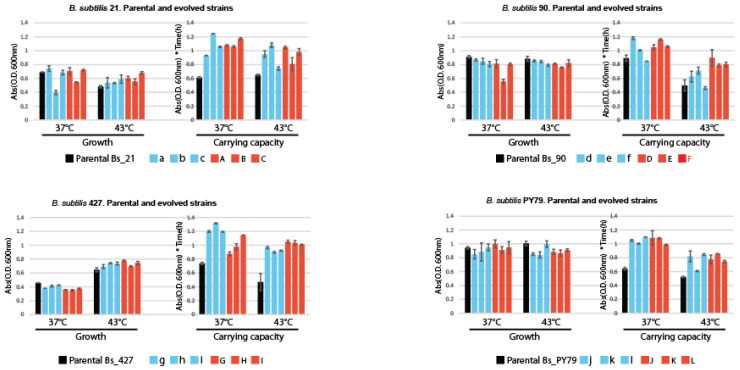
Growth and carrying capacity in parental and experimentally evolved lines.

Figure 4 shows the normalized results for *B. subtilis* strains. In Bs_21, significant changes were observed in both K37 and K43, suggesting substantial adaptation, particularly for the D-E-F lines under the high-temperature treatment. Bs_90 displayed pronounced effects in K43, specifically for the D-E-F lines, indicating a temperature-driven adaptation. However, the adaptation appeared limited, as growth improvements did not extend uniformly across all lines.

In Bs_427, we observed increases in both K37 and K43 values compared to the parental strain, reflecting an adaptation that may be driven by the growth medium. The enhanced carrying capacity at 43 °C suggests some degree of temperature adaptation, especially for the D-E-F lines. In Bs_PY79, all evolved lines showed increases in K37 and K43, consistent with medium-driven adaptation rather than temperature-specific changes.

Parental strains of *Bacillus subtilis* are represented by black bars, while their descendant lines under highly critical temperature (HCT) treatment are depicted with red bars and those under constant temperature treatment at 37 °C are shown in blue bars. Each panel comprises charts illustrating the maximum change in growth cultures of lines evolved at a constant low temperature (37 °C) and at a highly critical temperature (43 °C) on the left and carrying capacity (K) at 37 °C and 43 °C on the right.

#### 3.3.2. Impact of Temperature on Growth and Carrying Capacity on *Bacillus cereus* Lineages

**Figure 5:**
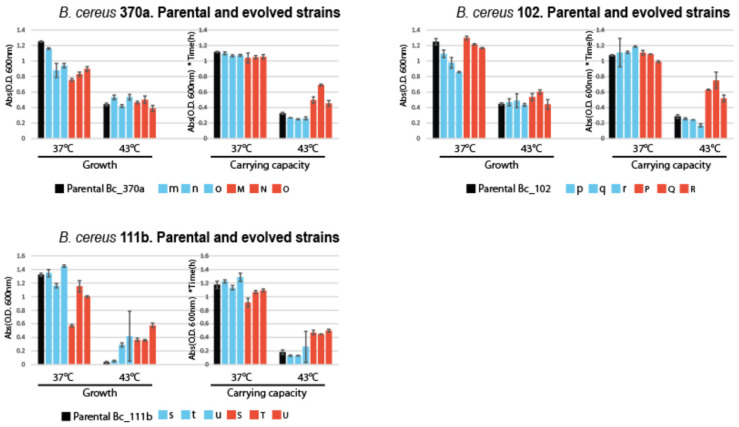
Growth and carrying capacity in parental and experimentally evolved lines.

Figure 5 illustrates the phenotypic changes in *B. cereus* strains. Bc_370a showed a strong effect on all lines for the parameter G37, implying adaptation to the growth medium rather than temperature. However, a significant impact on K43 was observed only in the M-N-O lines, which underwent high-temperature evolution, indicating some level of thermal adaptation.

In Bc_102, the analysis of K43 revealed stronger adaptation to high temperature in the P-Q-R lines compared to p-q-r. This suggests that thermal adaptation occurred despite the reaction norms not clearly indicating such changes. Specific growth rates (G37) also demonstrated some improvements, although these were less pronounced. For Bc_111b, G43 showed a marked increase across all evolved lines, indicating medium adaptation. However, K43 was significantly higher in the S-T-U lines compared to s-t-u and the parental strain, pointing to an evident effect of temperature adaptation.

Parental strains of *Bacillus subtilis* are represented by black bars, while their descendant lines under highly critical temperature (HCT) treatment are depicted with red bars, and those under constant temperature treatment at 37 °C are shown in blue bars. Each panel comprises charts illustrating the maximum change in growth cultures of lines evolved at a constant low temperature (37 °C) and at a highly critical temperature (43 °C) to the left and the carrying capacity (K) at 37 °C and 43 °C to the right.

Table 1 is a summary of the overall impact that evolution at either CTT or HCT had on the *B. cereus* and *B. subtilis* lineages. It is evident that a single line, Bs _427, was able to extend its thermal tolerance. None of the lines from the laboratory strain PY79 exhibited changes at any of the temperatures evaluated. All the evolved *B. cereus* lines reduced their growth capacity compared to the ancestral line.

##### 3.4. Changes in Colony Growth, Pigmentation, and Loss of Sporulation in Evolved Lines

We evaluated changes in colonial phenotypes by growing parental and evolved *Bacillus* strains on marine medium agar plates (Supplementary Figures S1 and S2). The top rows in the figures show the parental strains, while the lower rows display the six evolved strains (three evolved under the low-temperature treatment, LTT, and three under the highly critical temperature, HCT). Our semi-quantitative assessments revealed significant changes in colony growth among evolved strains. Notably, the *B. cereus*-evolved lines exhibited a substantial reduction in growth compared to their parental strains, a finding consistent with the reaction norm data. This reduction was particularly evident in lines M, N, Q, S, and T, which underwent HCT treatment. In contrast, the *B. subtilis*-evolved strains demonstrated more resilience, retaining a higher growth capacity.

The observed differences suggest a trade-off in *B. cereus*, where the selective pressure to endure high temperatures may have compromised their ability to grow rapidly. This trade-off likely represents an adaptive strategy prioritizing survival over growth efficiency. Specifically, the *B. cereus* strains appear to have evolved a mechanism to endure heat stress by reducing metabolic demands, which could explain their diminished growth rates.

Additionally, phenotypic changes were evident in colony pigmentation, especially among the *B. subtilis*-evolved lines. We observed distinct pigmentation changes in lines A, B, G, and I, indicative of altered metabolic or regulatory pathways as a response to prolonged heat exposure (Supplementary Figure S2).

We also noted a loss of sporulation ability in several evolved strains. This was particularly pronounced in the *B. cereus* HCT-evolved lines, suggesting that the evolutionary pressure to maintain vegetative cell function at high temperatures led to a trade-off, where sporulation capacity was compromised. This finding aligns with the hypothesis that *Bacillus* strains may sacrifice their ability to form heat-resistant spores to maintain active metabolic processes, which may be crucial for survival in fluctuating but non-lethal high temperatures.

### 3.5. Convergence in Mutations of Strains Evolved at Highly Critical Temperature Reveals the Role of di-C-AMP and Osmolytes in Temperature Tolerance

We sequenced the genomes of a representative clone from both the parental and evolved lines (seven parental genomes, 21 evolved genomes at CTT, and 21 evolved genomes at HCT). The genomes were assembled and annotated, and mutations were identified by comparing each evolved genome to its respective parental genome using GATK tools (see Materials and Methods). A workflow of Bioinformatic Analysis of Evolved Genomes and Mutations is shown in Supplementary Figure S3.

#### 3.5.1. Identification and Exclusion of Hypermutator Lines

**Figure 6:**
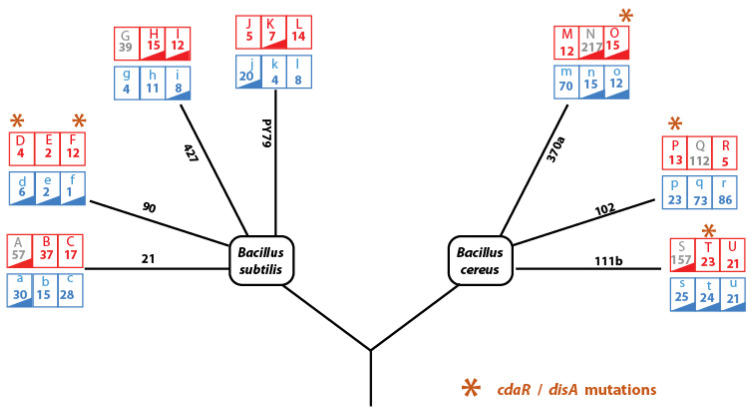
Mutation analysis of strains evolved from the *Bacillus subtilis* and *Bacillus cereus* lineages. Each lineage underwent highly critical temperature (HCT, red) treatment and constant temperature treatment (CTT, blue) with three replicates. The diagram illustrates the treatment conditions for each lineage and the total number of mutations observed after the genome sequencing of a colony from the final evolution transfer. Loss of sporulation in evolved lines occurred in strains marked with red or blue triangles. Lines in gray represent hypermutant phenotypes, which were excluded from our analyses to reduce noise in data correlation. Orange asterisks denote convergent mutations at the gene level in the cyclic d-AMP synthase gene (*cdaR* in *B. subtilis* or *disA* in *B. cereus*), found in independent evolving lines under highly critical temperatures, supporting evidence of positive selection (statistical analysis in the results section).

Seven lines exhibited hypermutator phenotypes, characterized by the accumulation of 39 to 219 mutations. These hypermutator lines were excluded from further analysis to avoid skewed results. The hypermutant criterion was established based on a significant deviation from the expected linear distribution of mutation accumulation (Supplementary Figure S5). Hypermutators were identified in both *B. subtilis* (lines A and G) and *B. cereus* (lines N, m, q, r, and S), with no apparent correlation to temperature treatment or bacterial lineage, suggesting that the hypermutant phenotype was independent of these factors. These hypermutant lines are depicted as gray labels in Figure 6.

#### 3.5.2. Classification of Mutations in Lines Evolved at 37 °C (CTT) and Above the HCT

**Figure 7:**
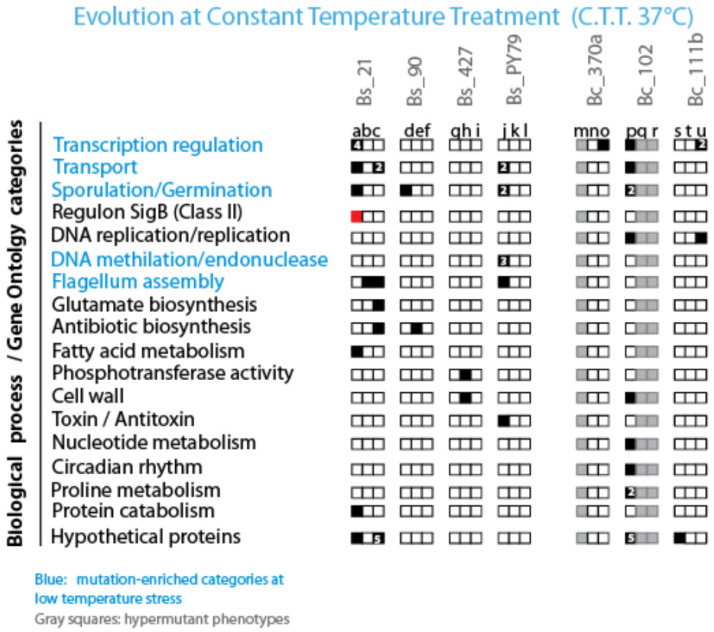
Distribution of mutations in lines evolved at constant low temperature (37 °C) across gene functional categories. The figure displays the distribution of mutations observed in lines evolved at a constant low temperature (37 °C), categorized by gene functional categories. Black squares indicate genes with at least one mutation in the depicted category, with the number within the black squares representing the number of mutated genes within that category. White squares denote the absence of mutations in the respective gene functional categories. Gray squares are used for all hypermutant strains that were not analyzed. Additionally, red squares highlight genes that have been described as being associated with the observed phenotype.

**Figure 8:**
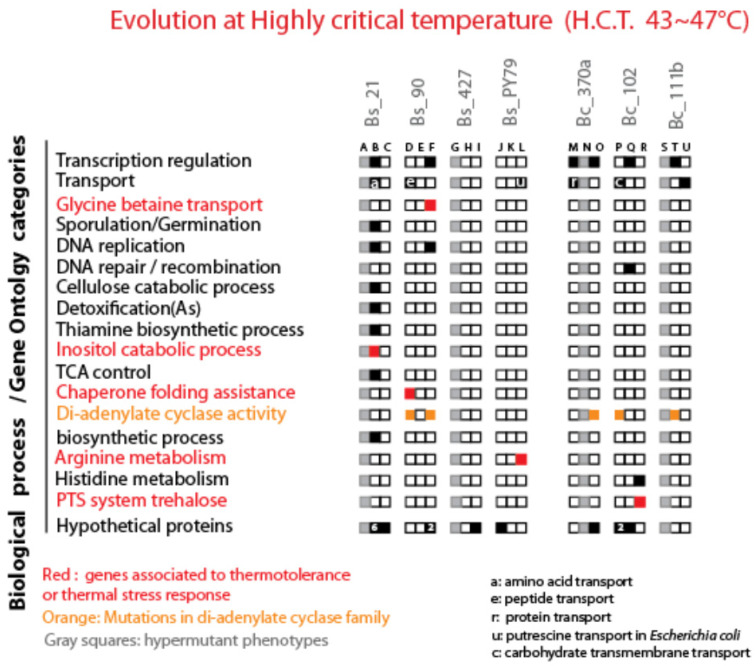
Distribution of mutations in lines evolved under highly critical temperature treatment (HCT) across gene functional categories. The figure illustrates the distribution of mutations observed in lines evolved under highly critical temperature treatment (HCT), categorized by gene functional categories. Black squares represent genes with at least one mutation in the depicted category, with the number within the black squares indicating the number of mutated genes within that category. White squares indicate the absence of mutations in the respective gene functional categories. Gray squares denote hypermutant strains that were not analyzed. Additionally, red squares highlight genes associated with the observed phenotype. An orange square specifically highlights di-adenylate synthase-encoding genes.

After excluding hypermutators, mutations were categorized into single-nucleotide substitutions, one to three base indels, and large genomic rearrangements, further classified as intergenic or intragenic. Most of the identified mutations were single-base indels, predominantly affecting open reading frames (ORFs). Figure 7 and Figure 8 illustrate the specific intragenic mutations. Supplementary Table S1 shows a list of all types of mutations in each evolved line. Sporulation loss and the quantification for each mutation type are shown in Supplementary Figure S6.

#### 3.5.3. Mutational Analysis in CTT-Evolved Lines

In CTT-evolved lines, we identified eight mutations in genes associated with transcription regulation (a-o-p-u lines), six mutations in transport-related genes (a-c-j-p lines), and six in sporulation-related genes (a-d-j-p lines). A significant mutation was detected in strain Bs_a within the SigB regulon, known for its role in thermal stress response [35]. Additional mutations were found in genes involved in DNA replication, methylation, flagellum assembly, metabolite biosynthesis, and cell wall structure (Figure 8).

#### 3.5.4. Mutational Analysis in HCT-Evolved Lines

In HCT-evolved lines, we found six mutations in transcription-regulation genes (B-F-M-O-Q-T lines) and six in transport genes (B-D-L-M-P-U lines), reflecting the impact on general metabolic processes. Only one mutation affected a sporulation-related gene, *lipC*, in line B of the Bs_21 strain without causing sporulation defects. Mutations of particular interest included those in glycine–betaine transport genes, crucial for both osmotic and thermal stress resistance [36]. Mutations interrupting the DnaD and RNase H encoding genes were found in line B of the Bs_21 strain. DnaD has been reported to be part of the replisome Turner 2004 and for replication fork progression under replication stress [37]. *B. subtilis* has two RNase H genes [38]. In bacteria, RNAse H is involved in the removal of RNA in RNA:DNA hybrids, and, in *B. subtilis*, the absence of one of the RNase H genes results in an accumulation of hybrids upstream of active coding genes with an impact in transcription and increased mutations [39]. Interestingly, strain Bs_B has 37 mutations, one of the most numerous in evolved lines (Supplementary Table S1). We also identified mutations in arginine metabolism genes linked to thermal stress in *B. subtilis* [35] and inositol metabolism genes known for their thermoprotective properties [40]. A chi-squared test comparing five thermal stress-associated mutations in HCT-treated lines to one in CTT-treated lines suggested a significant temperature-dependent effect.

#### 3.5.5. Uncovering of di-Adenylate Cyclases (DACs) in Temperature Adaptation

**Figure 9:**
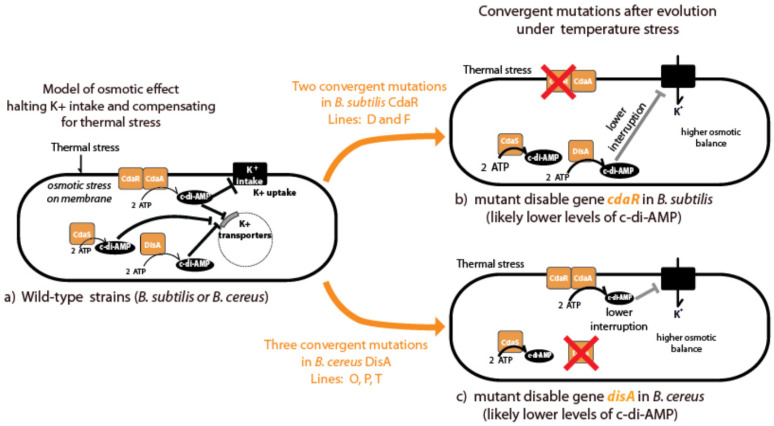
Convergent evolution in lines evolved at highly critical temperatures revealed cyclic di-AMP as a key player. The figure illustrates convergent evolution observed in lines evolved at highly critical temperature (HCT), highlighting cyclic di-AMP as a significant factor. A red X denotes the proteins predicted to be absent due to gene mutation. Both *Bacillus subtilis* and *Bacillus cereus* possess three cyclic di-AMP synthase genes, with two expressed during the vegetative phase and the third restricted to sporulation. In *B. subtilis*, the mutated gene was *cdaR*, a regulator of the synthetase gene, while in *B. cereus*, mutations impacted the *disA* gene, the synthetase itself. Cyclic di-AMP plays a crucial role in regulating K+ uptake, a critical factor in maintaining osmotic balance, suggesting its role in adapting to heat stress.

A noteworthy finding was the emergence of mutations in genes encoding di-adenylate cyclases (DACs), observed exclusively in HCT-evolved lines (Figure 9). DACs synthesize c-di-AMP from ATP, a secondary metabolite involved in growth regulation, membrane stability, glycine-betaine transport, and potassium uptake. We identified three loss-of-function mutations in the *disA* gene of *B. cereus* (lines O-P-T) and two in the *cdaR* gene of *B. subtilis* (lines D and F), with frameshifts occurring in the first half of the ORFs (Supplementary Figure S7). Binomial tests indicated a statistically significant occurrence of these mutations: a probability of 1.68 × 10^−4^ for *B. cereus* and 1.37 × 10^−3^ for *B. subtilis*. Given the total number of genes (5200 in *B. cereus* and 4100 in *B. subtilis*), these findings suggest a selective advantage for DAC-related mutations under high-temperature conditions.

## 4. Discussion

Our study highlights lineage-specific strategies by using experimental evolution with gradual temperature increases. It reveals significant differences in how *Bacillus subtilis* and *Bacillus cereus* lineages evolved thermal tolerance when subjected to gradual temperature increases beyond their thermal niche. *B. subtilis* strains displayed minimal changes in reaction norms, reflecting their greater inherent phenotypic plasticity, while *B. cereus* strains showed clear trade-offs, such as reduced growth rates at lower and even higher temperatures, indicating that high-temperature selection imposed a substantial evolutionary cost. These results suggest that larger initial plasticity might buffer against rapid adaptation under short-term experimental conditions.

In our study, the *B. cereus* strains initially struggled under high-temperature stress but managed to stabilize and recover growth within weeks, although, several times, we had to stop the regime of increasing temperature until the final 300 generations of constant high-temperature selection. These strains accumulated more mutations than those of the *B. subtilis* lineage. This is similar to what Mongold et al. [28] observed for *E. coli* growth at the limit of their niche temperature and suggests that evolutionary rescue mechanisms were at play, allowing the strains to persist despite the harsh conditions. The concept of evolutionary rescue is particularly relevant to our observations. Evolutionary rescue occurs when adaptive genetic changes enable populations to recover from near extinction under severe environmental stress [41,42].

While *B. subtilis* benefited from its broad thermal tolerance, *B. cereus* needed substantial genetic adjustments to cope with high temperatures. This observation raises critical questions about the role of plasticity in evolutionary dynamics. Does higher plasticity hinder adaptation by reducing selective pressure, or does it enable immediate survival, allowing for gradual genetic changes? Our data support the notion that short-term evolution favors species with inherent plasticity, while long-term selection can drive adaptive genetic changes in species with less initial plasticity. *B. subtilis* can be considered to possess more thermal plasticity compared to *B. cereus*. This conclusion aligns with previous findings by Hurtado et al. [14], which highlighted differences between the *B. cereus* and *B. subtilis* lineages in their upper tolerance limits, comparing strains from a thermal spring to those from a temperate lagoon. In this study, only *B. subtilis*-evolved lines exhibited better growth than the parental strain at the HCT. At the same time, three of four *B. subtilis* lineages surpassed their ancestral strain’s tolerance limit even at 49 °C. However, it is essential to note that the *B. subtilis* lines evolved at 37 °C were also able to increase their thermal limit. The pressure of maintaining continuous growth in this particular medium impacted the metabolism that favored thermal tolerance. This is concordant with the change in carrying capacity that resulted from the experimental evolution, particularly for the *B. subtilis* lines, for which higher carrying capacity changes occur in all lines, whether evolved at 37 °C or at high temperature. This contrasts with *B. cereus* lines, for which only the lines evolved at higher temperatures exhibit increased carrying capacity under heat stress. These results suggest a shift in metabolism. Accordingly, the expression of proteins that provide stress tolerance or the capacity to adapt to new environmental conditions rapidly is expected to be at the expense of growth-related proteins, reducing the instantaneous growth rate [43].

Finally, adaptation at high temperatures came with trade-offs, as evidenced by poorer growth at lower temperatures. This was observed for *B. cereus* lines but not for *B. subtilis*. Our results showed decreased fitness of some of the evolved *B. cereus* lines (Bc111 and Bc370) at what was the ancestral strain’s optimal growth temperature, as a trade-off for adapting to high temperatures. This supports evolutionary theory, which suggests pleiotropic effects, where genes affecting multiple traits may constrain adaptation and result in maladaptation [44].

Genetic analysis revealed that mutations in genes involved in osmolyte metabolism and heat-shock responses played critical roles in thermal tolerance. For instance, arginine and trehalose metabolism mutations were associated with improved thermal resilience. Arginine biosynthesis is known to be induced under heat stress [35], and trehalose has been suggested to act as a thermoprotectant, stabilizing proteins and membranes [45]. These mutations differ from those described in Ref. [46] from an experimental evolution experiment with *B. subtilis*, with a temperature regime of 50 °C. Sequencing revealed mutations in genes encoding proteins associated with pathways known to be involved in heat stress response (*hrcA*, *ftsH*) and other stress proteins (*relA* and *sigW*). We do not expect to find mutations in genes involved in the thermal response, such as those described by Hecker et al. (1996) [36] and Helmann et al. (2001) [35], which, if mutated, would be expected to reduce tolerance to temperature.

A novel and significant finding of our study was the convergence of mutations in DAC genes in several strains in both lineages. DAC genes are involved in the synthesis of c-di-AMP. Both *B. cereus* and *B. subtilis* possess three genes for the synthesis of c-di-AMP, DisA, and CdaA function in the vegetative phase, while CdaS is sporulation-specific [47]. Most studies have been carried out in *B. subtilis*, and it is known that the activity of CdaA is modulated by CdaR. c-di-AMP is essential for the growth of *B. subtilis* and DisA and CdaA contribute to modulating different DNA damage responses during exponential growth [48]. The parallel occurrence of mutations in DAC genes (*cdaR* in *B. subtilis* and *disA* in *B. cereus*, Figure 9) is intriguing. Several lines of evidence suggest that the di-adenylate cyclase CdaA is part of the conserved essential *cda-glm* module involved in cell wall metabolism. Notably, this molecule regulates potassium transport and osmotic balance [49]; when bacteria are challenged with high osmolarity, they acquire increased resistance to high temperature and oxidative stresses. The high-osmolarity-dependent increase in thermotolerance has two manifestations: the elevation of the upper limit of the growth temperature and enhanced survival at otherwise lethal high temperatures [50]. Although c-di-AMP is involved in many essential pathways, to participate in virulence and osmotic stress, it has not been implicated in thermal stress. The parallel occurrence of mutations in DAC genes (*cdaR* in *B. subtilis* and *disA* in *B. cereus*, Figure 9) strongly suggests its role in heat stress tolerance. Our results suggest a lineage-specific evolution in the modulation of the c-di-AMP regulation. In either case, potassium uptake is likely involved in mitigating membrane destabilization caused by high temperatures. In *B. subtilis* and many other bacteria, a sudden increase in the intracellular potassium concentration is the first response to osmotic stress [51]. Thus, the control of potassium homeostasis by c-di-AMP might also be part of a larger picture of the regulation of osmoadaptation. It has been shown that a variety of compatible solutes (i.e., glycine–betaine, proline) serve as heat protectants for *B. subtilis*. *B subtilis* has several redundant transporters, such that a mutation is one of the transporters that has no impact on heat stress [52] on molecular adaptation regardless of whether it restores physiological and molecular processes from a stressed state back toward the unstressed, wild-type state, or whether it instead tends to drive the evolution of novelty [53]. Previous studies have suggested the former because studies have shown that *E. coli* adapts to high-temperature stress (42.2 °C) by restoring both gene expression [54,55] and phenotypic characteristics [27] toward that of the unstressed ancestor. We think that expanding the thermal niche requires both adapting gene expression to a new threshold, as the previous level is inadequate for the permanent extra-limit temperature stress and the novelty to withstand permanent stress. In this sense, we suggest that cells regulate the levels of c-di-AMP to fit the novel stressful conditions. These findings open up new avenues for understanding the role of c-di-AMP in bacterial heat stress response.

Our experimental design, starting with two species and multiple strains, allowed us to explore the potential for parallel evolution [56] and identify multiple pathways leading to thermal tolerance. Parallel evolution, defined as the independent evolution of the same phenotype or genotype in response to identical selection pressures, provides insights into the predictability and diversity of evolutionary outcomes. In this study, we observed that while mutations affecting c-di-AMP synthesis emerged in all three *B. cereus* lineages, only one *B. subtilis* lineage (Bs_90) evolved a similar mutation. This finding suggests that while the modulation of c-di-AMP synthesis is a common and potentially “go-to” solution for thermal tolerance, it is not the only pathway. The presence of c-di-AMP mutations in multiple lineages across species highlights its broad utility, yet the absence of these mutations in some evolved lines underscores the diversity of evolutionary strategies. This observation supports the notion that standing genetic variation provides multiple routes to equivalent phenotypes, with natural selection shaping the most accessible solutions within specific genetic and physiological contexts.

Multiple pathways can lead to thermal tolerance, even under identical selective pressures, emphasizing the power of parallel evolution to reveal evolutionary constraints and opportunities. These findings align with the idea that evolution often proceeds along a single pathway within a given lineage but that alternative routes remain viable under different genetic or environmental conditions. By leveraging two species and multiple strains, our study underscores the importance of comparative approaches in experimental evolution for uncovering shared and lineage-specific adaptation mechanisms.

Our findings suggest that while different genetic pathways can lead to thermal tolerance, these pathways may converge on similar metabolic routes, such as osmotic protection. Mutations in genes regulating c-di-AMP synthesis highlight one such path, given its role in potassium transport and osmotic balance under stress. However, it is important to acknowledge a significant caveat in our analysis: genome sequencing identifies intragenic mutations that could be associated with the phenotype but cannot infer the impact of mutations in intergenic regions. Additionally, when multiple mutations occur within a genome, it is challenging to discern which specific mutations are responsible for the observed phenotypes. When working with wild strains, it is more challenging to reintroduce single mutations to validate the phenotype. Transcriptomic analyses would be necessary to uncover the overarching metabolic strategies and determine how these mutations integrate into the broader cellular response to thermal stress. Such analyses could reveal differential gene expression patterns and regulatory networks contributing to adaptation, providing a more comprehensive understanding of the metabolic adjustments underlying thermal tolerance.

The limited thermal tolerance observed in our study, even after experimental evolution, raises concerns about the capacity of microbial communities to adapt to climate change. Given the vital roles of *Bacillus* species in biogeochemical cycles, constraints on their thermal adaptability could have cascading effects on ecosystem functions, nutrient cycling, and soil health. Our findings suggest that while some bacterial populations may experience evolutionary rescue, the associated trade-offs could limit their ecological success and impact global processes.

It is notable that the three strains within each *Bacillus* lineage (*B. subtilis* and *B. cereus*) exhibited consistent, lineage-specific outcomes. Furthermore, the three independently evolved lines for each strain displayed similar trends in temperature tolerance, despite differences in the underlying mutations driving their responses. This result suggests a strong influence of both genetic background and lineage-specific traits in shaping the adaptive responses to temperature stress in *Bacillus* species. The consistency in outcomes within each lineage (*B. subtilis* and *B. cereus*) highlights that certain inherent genetic or physiological characteristics predispose these lineages to follow specific evolutionary trajectories.

Our observations indicate that *B. cereus* lineages tended to accumulate more mutations yet did not achieve markedly better fitness at higher temperatures, whereas *B. subtilis* lineages adapted to elevated temperatures with comparatively fewer mutations, suggesting greater inherent thermal plasticity or more efficient physiological and regulatory mechanisms for coping with thermal stress. Several factors may underlie these differences. First, *B. subtilis* may possess a more robust stress response system, including efficient chaperones, heat-shock proteins, and membrane adaptations that minimize the need for extensive genetic change. Second, differences in metabolic versatility and regulatory network flexibility may enable *B. subtilis* to rapidly optimize gene expression and protein function at higher temperatures, while *B. cereus* may rely more on genetic alterations that do not directly translate into fitness gains. Additionally, species-specific genomic architecture and mutation biases could shape distinct evolutionary trajectories, with *B. cereus* potentially having less efficient DNA repair or a mutational spectrum that fails to confer adaptive benefits under thermal stress. Furthermore, the evolutionary histories and ecological niches of these species influence their baseline adaptability; for instance, the laboratory-adapted *B. subtilis* strain-like PY79 may have evolved pre-adaptations suited to fluctuating conditions, whereas *B. cereus*, adapted to more stable environments, struggles to exploit novel thermal niches despite generating substantial genetic variation. For example, such lineage-specific adaptations have been documented by Guinebretière et al. (2008) [57], who showed that different taxonomic groups of *B. cereus* display lineage-associated thermal profiles ranging from 7 to 50 °C, explaining why *B. cereus* populations in warm parts of a dairy process differ from those in colder parts [58].

The observed lineage-specific outcomes and convergence within strains underscore the critical role of genetic background in shaping adaptive responses. However, the diversity of underlying mutations leading to similar phenotypic outcomes highlights the unpredictability of evolutionary pathways. This variability suggests that microbial responses to environmental stressors, such as climate change, may be highly context-dependent and difficult to generalize. Each genetic background provides a distinct set of constraints and opportunities, leading to diverse adaptive trajectories even under similar selective pressures.

Consequently, this result makes it more challenging to predict how microbial communities, composed of diverse genetic lineages, will respond to global warming. While some lineages may adapt via predictable mechanisms, others may follow entirely different and unforeseen evolutionary paths. This underscores the need for further studies that explore the interplay between genetic background, evolutionary dynamics, and environmental change to better understand and anticipate microbial responses at both the species and community levels.

In addition to well-characterized experimental evolution studies in *E. coli* (e.g., Tenaillon et al. [24]), recent research has increasingly explored evolutionary dynamics in *Bacillus* and other wild bacterial lineages under various selective pressures. For instance, *B. subtilis* has served as a model in experimental evolution to investigate adaptation in plant-associated environments and in the presence of competing species. Pomerleau et al. [59] demonstrated that *B. subtilis* rapidly evolved enhanced root colonization capacity under biotic stress imposed by *Pseudomonas* species, pinpointing mutations in regulators linked to biofilm development. Likewise, Blake et al. [60] showed that *B. subtilis* populations adapted to the rhizosphere of *Arabidopsis thaliana* diversified into distinct lineages, with some evolved strains improving their host-specific colonization abilities. These findings highlight how environmental context shapes adaptive trajectories and can promote genetic diversification.

Other studies emphasize the role of genetic mechanisms, such as horizontal gene transfer (HGT), in shaping adaptation. Slomka et al. [61] found that *B. subtilis* populations evolving under high-salt conditions acquired extensive foreign DNA, improving salt tolerance via bursts of HGT. Although our study focused on thermal adaptation rather than osmotic stress, these results underscore the importance of both point mutations and gene acquisition in driving evolutionary responses and raise the question of whether thermal adaptation might also draw on similar genetic sources if available.

Environmental fluctuations and life-history traits can further influence evolutionary outcomes. Shoemaker et al. [62] reported that the capacity for dormancy (“seed banking”) in *B. subtilis* alters the trajectory of molecular evolution over extended timescales, increasing genetic diversity and affecting the direction of selection on certain genes. Although our experimental design did not directly examine dormancy states, such findings suggest that the intrinsic traits of species, including the ability to form spores, may shape the pace and path of thermal adaptation.

Finally, recent molecular and genomic studies highlight the complexity of responses at the RNA and gene-regulatory levels. For example, Jolley et al. [63] demonstrated that *B. subtilis* dynamically adjusts its RNA structure in response to temperature changes, potentially providing an additional layer of rapid, non-mutational adaptation. Similarly, Hawkins et al. [64] and Shi et al. [65] explored how essential genes and sporulation-related loci evolve over time, offering insights into how baseline genomic architecture and gene regulatory networks can influence adaptive potential.

Taken together, these studies show that *Bacillus* lineages can evolve through multiple genetic routes—ranging from single-nucleotide changes and HGT events to alterations in regulatory networks—to cope with diverse selective pressures, including thermal stress. They also reveal common themes, such as the role of regulatory mutations and pre-existing physiological traits, which can set certain lineages on more efficient adaptive trajectories. Integrating this broader perspective into our findings suggests that while the mutation rate and the spectrum of mutations we observed may not necessarily correlate with thermal niche expansion, they reflect a complex interplay of genetic background, regulatory flexibility, ecological context, and evolutionary history. By situating our results alongside these other *Bacillus* and bacterial evolution studies, we gain a more nuanced understanding of how constraints and contingencies influence the adaptive landscape of bacterial populations facing environmental challenges.

The experimental constraints of this study, which included the passes for only 1000 generations and laboratory-based conditions, may not fully reflect the complexities of long-term evolutionary dynamics or the ecological interactions that occur in natural settings. Additionally, while we identified several mutations, especially those related to di-adenylate cyclase (DAC) genes, more studies would be necessary to understand their roles. This could involve transcriptomic analysis to better understand gene expression patterns in response to thermal stress. Furthermore, exploring the broader ecological context of these findings, such as how they translate to natural microbial populations and how bacteria respond to other environmental stressors like desiccation or nutrient scarcity, will be essential to evaluate bacterial adaptability and resilience comprehensively.

## 5. Conclusions

Our results demonstrate that *Bacillus* strains exhibit limited thermal adaptation and are lineage-specific, with immediate trade-offs even under prolonged selective pressure. This constrained evolvability poses significant implications for microbial responses to global warming, potentially affecting biogeochemical cycles and ecosystem stability. Understanding these evolutionary limits is crucial as we prepare for the ecological impacts of climate change.

This study emphasizes the need for more research into the evolutionary and ecological limits of microbial adaptability to predict and mitigate the broader consequences of climate change on vital biogeochemical processes.

## Figures and Tables

**Table 1:** Summary of temperature plasticity gained on growth and carrying capacity by the evolved lines.

	Growth										Carrying Capacity (K)			
Evaluated at:	27 °C		37 °C		43 °C		46 °C		49 °C		37 °C		43 °C	
Strain evolved at:	37 °C	HCT	37 °C	HCT	37 °C	HCT	37 °C	HCT	37 °C	HCT	37 °C	HCT	37 °C	HCT
Bc 370a *	▼	▼	▼	▼	▼	▼		▼	▼	▼				▲
Bc 11b *		▼		▼		▲		▼		▼		▼		▲
Bc 102 *	▼	▼	▼		▼	▲			▼	▼				▲
Bs 21			▲	▲	▲	▲			▲	▲	▲	▲	▲	▲
Bs 90 *			▼	▼	▼	▼			▲	▲	▲	▲	▲	▲
Bs 427					▲	▲	▲	▲	▲	▲	▲	▲	▲	▲
BS PY79		▼									▲	▲	▲	▲

▼ Lower capacity than ancestral line. ▲ Higher capacity than the ancestral line. Empty squares mean the same or similar capacity than the ancestral line. Red, evolved at temperatures above highly critical temperature. Blue, evolved at 37 °C. * Points to strains where some lines had mutations in c-di-AMP synthesis genes.

## Data Availability

The strains are available upon request to interested researchers.

## Supplementary Materials

The following supporting information can be downloaded at: https://www.mdpi.com/article/10.3390/biology13121088/s1. Supplementary Figure S1A,B. Recovery Ability of *B. subtilis* and *B. cereus* Strains After Incubation at High Temperatures. Supplementary Figure S2. Growth phenotypes on semisolid medium of Evolved *B. subtilis* and *B. cereus* Strains at High Temperature. Supplementary Figure S3. Workflow of Bioinformatic Analysis of Evolved Genomes and Mutations. Supplementary Figure S4. Principal Component Analysis (PCA) of Experimental Evolution Treatments. Supplementary Figure S5. Criterion for Classification of Hypermutant Phenotypes. Supplementary Figure S6. Sporulation Loss and Mutation Type Quantification. Supplementary Figure S7. Open reading frame analysis of intragenic mutations in selected genes. Supplementary Table S1. Raw Data for Mutation Counts and Sporulation Loss (Excel table). Supplementary Table S2. Evolved lines and chosen regions re-sequenced to validate mutations and Oligonucleotides used for the verification of Mutations. Supplementary Table S3. Mutations validated by PCR amplification and sequencing.
