# Supplementary material for: Thermal Plasticity and Evolutionary Constraints in Bacillus: Implications for Climate Change Adaptation
Source: Biology (Basel). 2024 Dec 23;13(12):1088. doi: 10.3390/biology13121088 (PMC11673879; doi:10.3390/biology13121088)
Supplement: Supplementary file 1 [file biology-13-01088-s001.zip › Dec 16th Supplementary Figures and Tables Hurtado_Bautista et al et al 18 Nov 2024 FINAL.pdf]

Type of the Paper *Article*

Thermal Plasticity and Evolutionary Constraints in *Bacillus*: Implications for Climate Change Adaptation

Enrique Hurtado-Bautista [1], Africa Islas-Robles [1], Gabriel Moreno-Hagelsieb [2] and Gabriela Olmedo-Alvarez [1,*]

[1] Departamento de Ingeniería Genética, Unidad Irapuato, Cinvestav 36824, Mexico; enrique.hurtado@cinvestav.mx (E.H.-B.); africa.islas@cinvestav.mx (A.I.-R.)
[2] Department of Biology, Wilfrid Laurier University, Waterloo, ON N2L 3C5, Canada; gmoreno@wlu.ca
* Correspondence: golmedo@cinvestav.com

Supplementary Material:

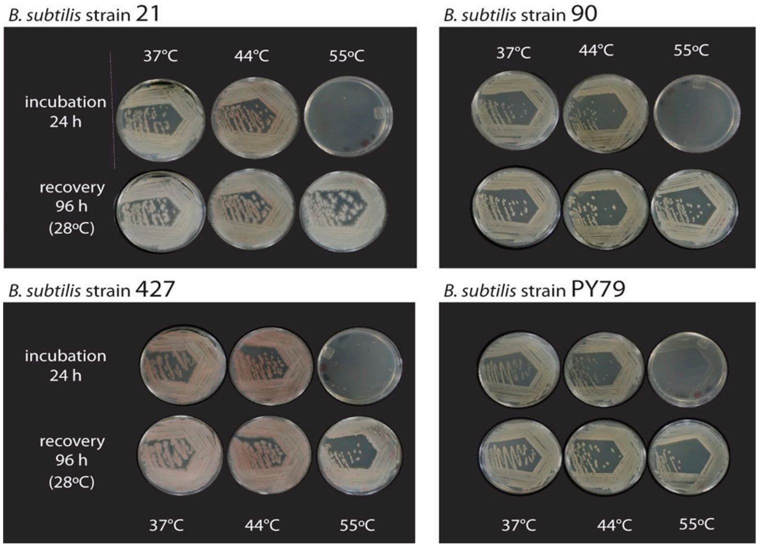

(**A**)

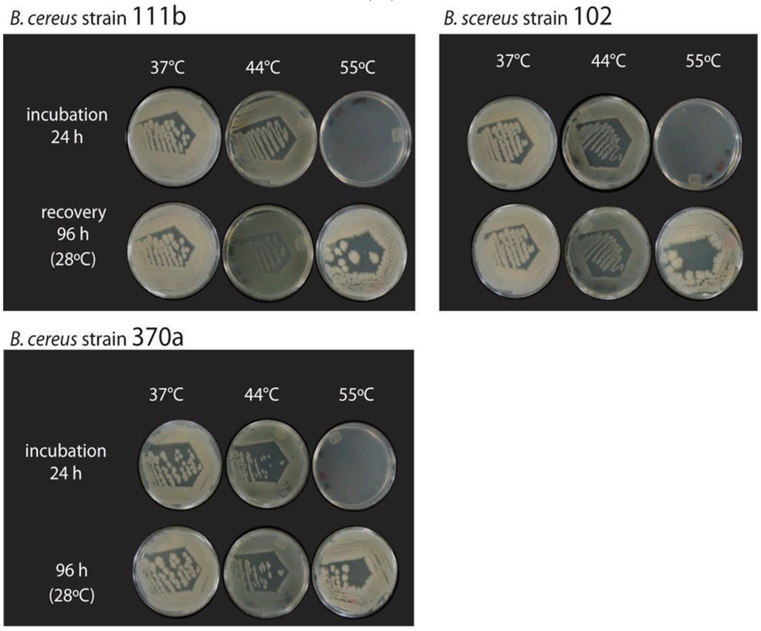

(**B**)

Supplementary Figure S1. A. Recovery Ability of *B. subtilis* Parental Strains After Incubation at High Temperatures. Photographs depict colony growth of *B. subtilis* strains on marine medium agar after 24 hours of incubation at the respective temperatures, followed by a 96 h recovery period at 28°C. No colonies were observed at 55°C during initial incubation, but after transfer to 25°C, all plates exhibited normal colony growth, showing the resilience of *B. subtilis* strains under extreme heat stress. **B**. Recovery Ability of parental *B. cereus* Strains After Incubation at High Temperatures. Photographs depict colony growth of *B. cereus* strains on marine medium agar after 24 hours of incubation at the respective temperatures, followed by a 96 h recovery period at 28°C. No colonies were observed at 55°C during initial incubation, but after transfer to 25°C, all plates exhibited normal colony growth, showing the resilience of *B. subtilis* strains under extreme heat stress.

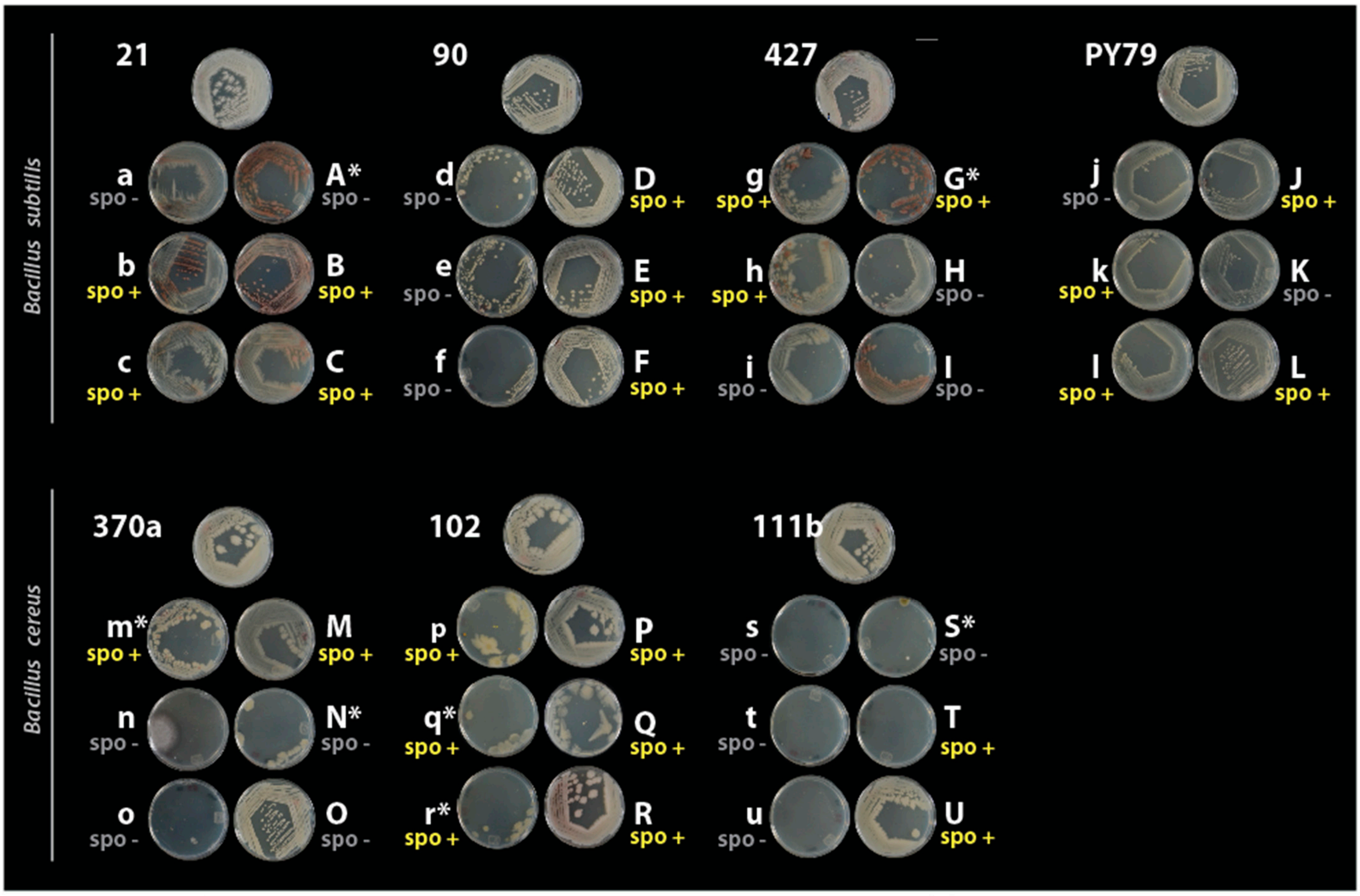

Supplementary Figure S2. Changes in Colony Growth, Pigmentation, and Loss of Sporulation in Evolved Lines

We evaluated changes in colonial phenotypes by growing parental and evolved *Bacillus* strains on Marine medium agar plates (Supplementary Figures S1 and S2). The top rows in the figures show the parental strains, while the lower rows display the six evolved strains (three evolved under the Low-Temperature Treatment, LTT, and three under the High Critical Temperature, HCT).

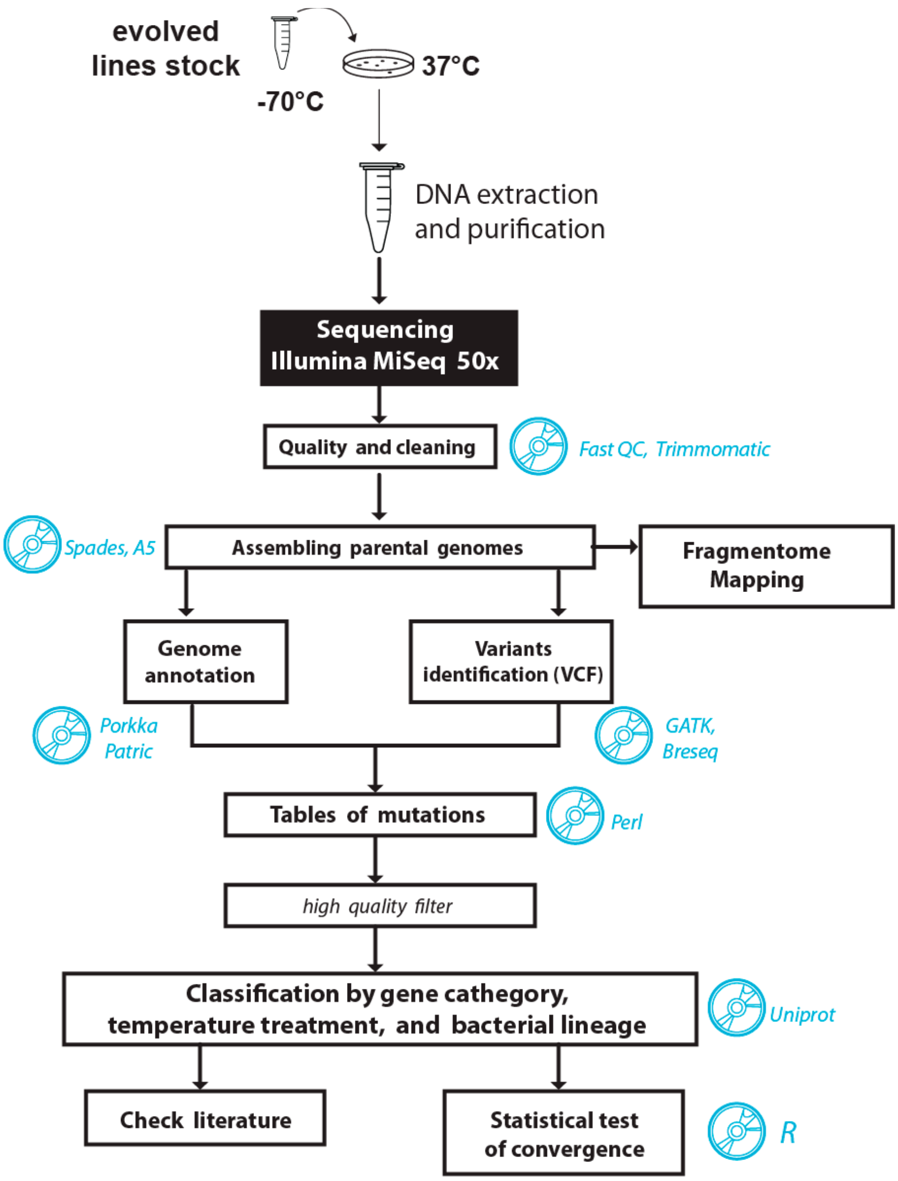

Supplementary Figure S3. Workflow of Bioinformatic Analysis of Evolved Genomes and Mutations. Step-by-step bioinformatic workflow used for analyzing the genomes of evolved lines and identifying mutations. The process included genome sequencing, quality control, read alignment to the reference genome, variant calling, annotation of mutations, and their classification into functional categories. This workflow highlights the computational pipeline utilized to uncover genetic changes associated with thermal adaptation in the evolved *Bacillus* strains.

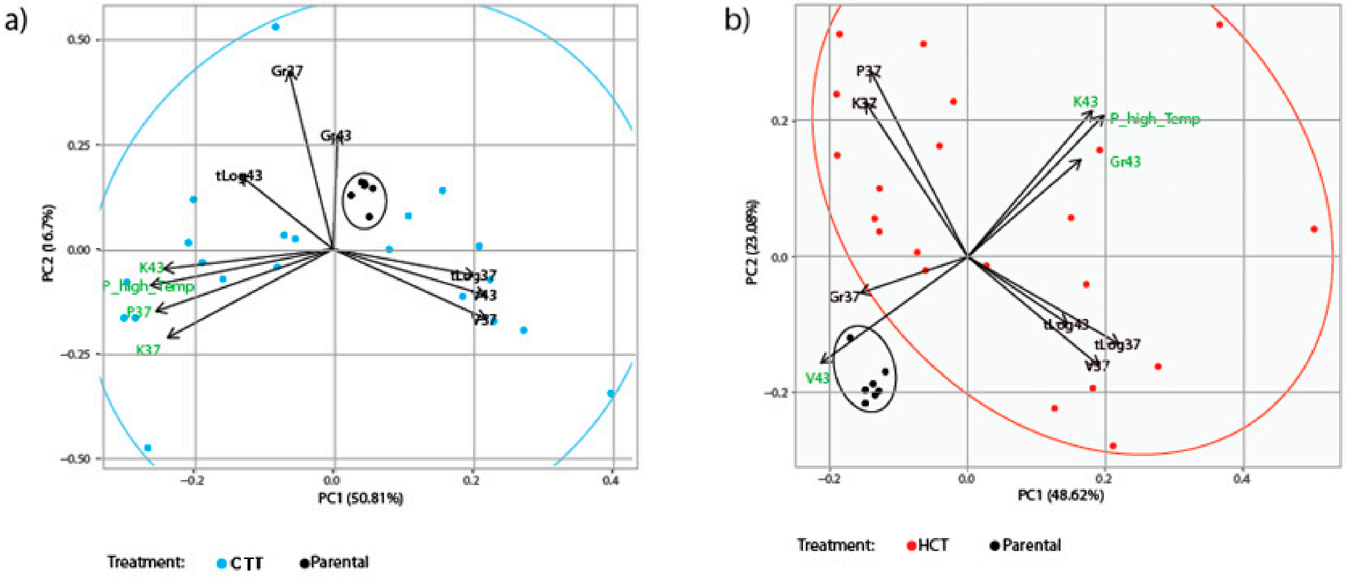

Supplementary Figure S4. Principal Component Analysis (PCA) of Experimental Evolution Treatments. The PCA of two experimental evolution treatments: evolution at 37°C (blue, left) and at high critical temperature (HCT, red, right). Vectors represent the direction and magnitude of changes in key growth and performance features after 1,000 generations of serial transfers. Features include growth rate (Gr), total carrying capacity (K, maximum optical density), velocity of exponential phase onset (V), performance (P, area under the OD-temperature function), and the duration of the exponential phase (tLog). These features were evaluated at both 37°C and 43°C, highlighting the phenotypic responses and adaptations under different temperature regimes.

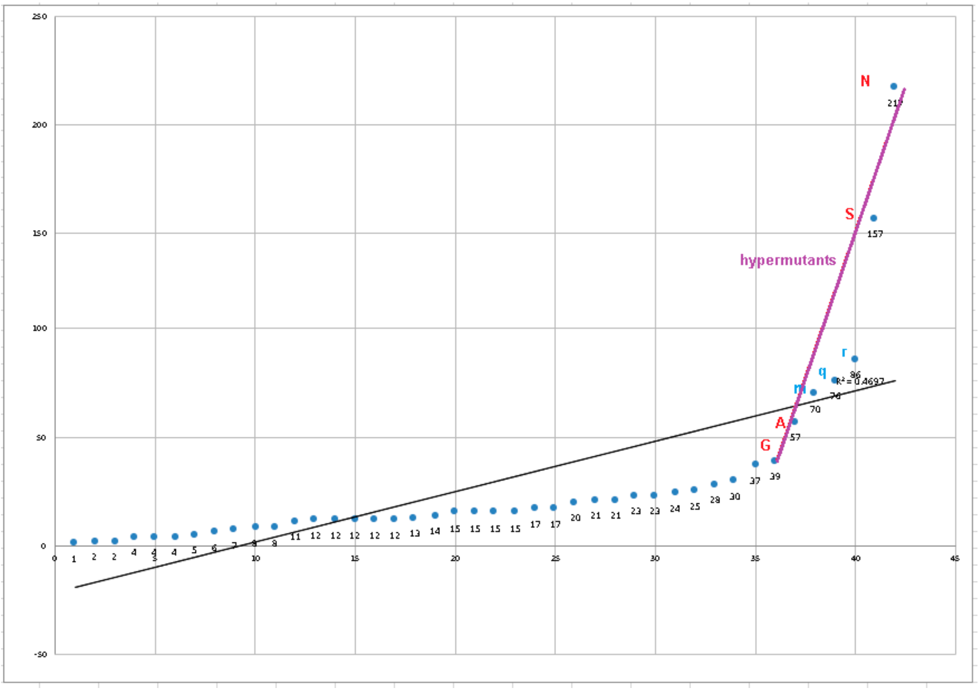

Supplementary Figure S5. Criterion for Classification of Hypermutant Phenotypes. The criterion used to classify hypermutant phenotypes based on the number of mutations identified in 42 sequenced strains. Mutations were plotted in sequential ascending order, and a linear function was applied across the data. A gradual increase in mutations was observed from 1 to 39, followed by a sharp rise in the final seven genomes, where mutation counts ranged between 57 and 200. Strains in this latter group were classified as hypermutants, as their mutation rates deviated significantly from the linear trend observed in the majority of the strains.

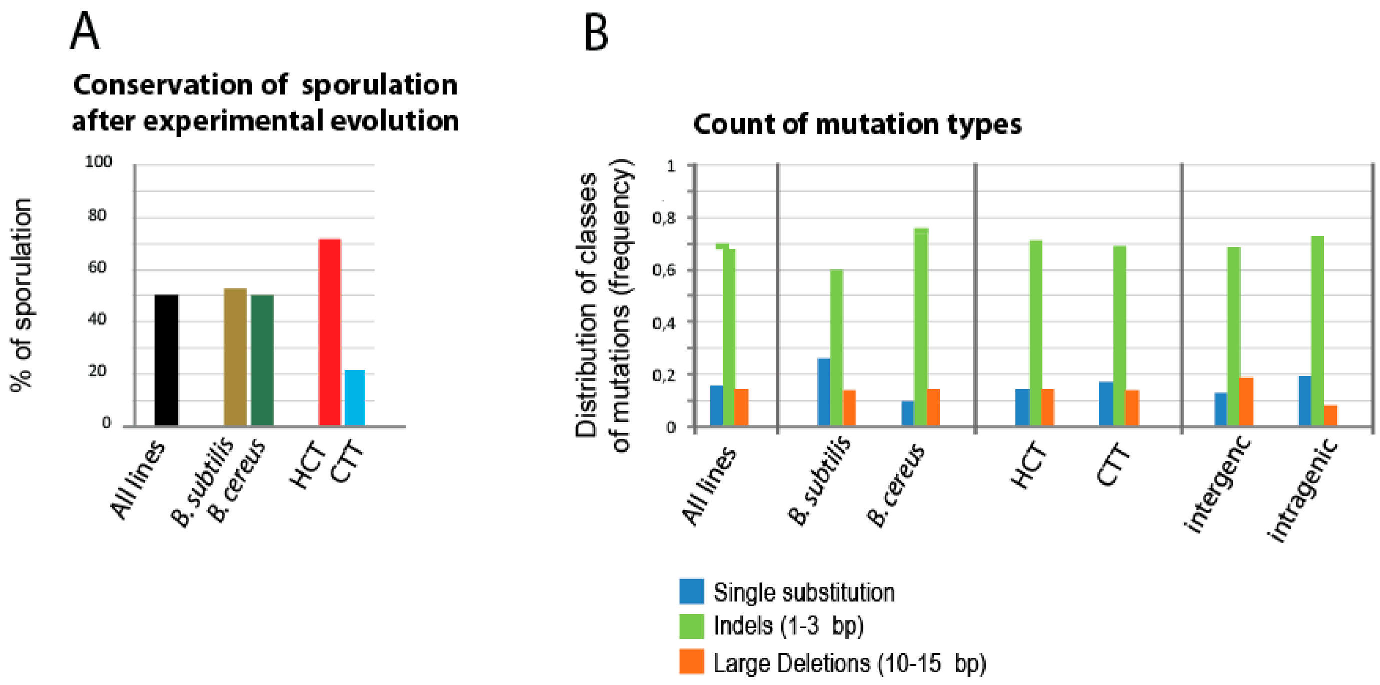

Supplementary Figure S6. Sporulation Loss and Mutation Type Quantification (A) Quantification of sporulation loss after experimental evolution, analyzed by lineage (*B. cereus* and *B. subtilis*) and experimental treatment (HCT: high critical temperature or CTT: constant temperature at 37°C). The Y-axis represents the percentage of strains retaining sporulation capacity. (B) Quantification of mutation types across groups categorized by lineage, treatment temperature, and whether mutations were intragenic or intergenic. This analysis highlights lineage- and treatment-specific differences in the genetic changes associated with experimental evolution.

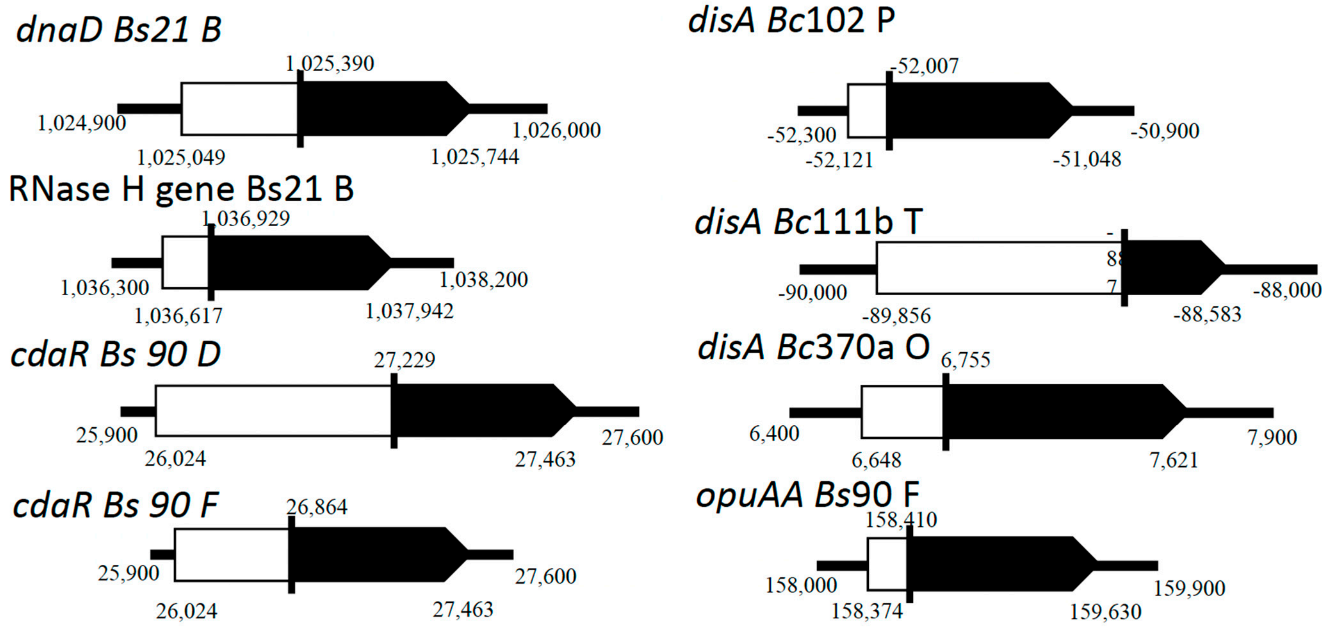

Supplementary Figure S7. Open reading frame analysis of intragenic mutations in selected genes.

An analysis was conducted to detect the open reading frame (ORF) corresponding to the gene of interest. For each gene, this analysis is depicted in a series of figures. The figure displays the ORF as an open arrow pointing in the direction of the gene, from start to end. This validates the

presence of the mutation within the gene of interest (intragenic mutation) and shows the position at which the reading frame is interrupted (see Supplementary Table S3).

Supplementary Table S1. Raw Data for Mutation Counts and Sporulation Loss. (separate Excel Table)

Raw data for mutation counts, including their genomic location, and observations on the loss of sporulation across all experimental lines. The data include non-validated mutations in rRNA elements, which may represent false positives due to the multiple copies of rRNA genes in the genome. This comprehensive dataset supports the analysis of mutation types and their potential impacts on phenotypic traits such as sporulation capacity.

Supplementary Table S2. Evolved lines and chosen regions re-sequenced to validate mutations

Parental strain	Evolved line	Mutation	Implicated gene product or vicinity	Forward oligo	Reverse Oligo
Bc_370a	M47	Mut. 1	Protein translocase subunit SecA	TGTAAGGCAATACCACCCATT	GGGAAATACGGTTTAATCGAAA
Bc_102	P47	Mut. 2	Protein SapB	ACTCAATCCATTCGCTCGAC	CGTTCTAAACCAATGGCAAA
Bc_102	Q47	Mut. 3	DNA-invertase hin	TTTGTTTCTTCTTCTGCGAGAG	CATAAGGGCGATACGGCTAC
Bs_21	B47	Mut. 4	Citrate synthase 2	CGATTTTAGCCGTCGCTTTA	TAGTGGCGACTACCCCTTCA
Bs_21	B47	Mut. 5	Endoglucanase S	CCGATGTTCAGCAAGCTTTA	TGAATGTTCATTCCGCAGAT
Bs_21	B47	Mut. 6	Spore germination lipase LipC	ATCGAAAAATTTGCGCTGAA	AACGGCATAGTTTTTAATCGTGA
Bs_90	F47	Mut. 7	hypothetical protein PF09954	GCGTTCTTCTTCTACATTTTTGTG	TCTGAGGTCACCGTAACCAA
Bs_21	B47	Mut. 10	DNA replication protein DnaD	GCTGGGCCTTAATGAGACAGA	GCGGTCAATGTAGCGAAAAT
Bs_21	B47	Mut. 11	RNase_H superfamily (domain)	ACAAAGAATGGGCTGCACTC	TTTTAACCTGAGGCCAATCGA
Bs_90	D47	Mut. 12	CdaA regulatory protein CdaR	GGATTTTGACGGAGTTTCCA	TCGCTCCTTTTTCATTCTCA

Bs_90	F47	Mut. 13	CdaA regulatory protein CdaR	TGTGGATGTTGAGCCATCAGT	GGGAGAACCTTTTGCAGTGA
Bc_370a	O47	Mut. 14	DNA integrity scanning protein DisA	CTTCGCTACACGTTCTGCTGT	CGGATGGGATTGAGGTTGTA
Bc_102	P47	Mut. 15	DNA integrity scanning protein DisA	CGGATGGGATTGAGGTTGTA	CTTCGCTACACGTTCTGCTGT
Bc_111b	T47	Mut. 16	DNA integrity scanning protein DisA	ATGGCAAAAATTCAGGCAAG	ACCAACATGCCGCTTTTATC
Bc_102	Q47	Mut. 17	Protein RecA -	GGATTGGCGATTAAAGGTGA	ATAATACGGCCACGTGGGTA
Bs_90	F47	Mut. 18	Glycine betaine transport ATP-binding protein OpuAA	CCGATGATATTACCTGGAAAGCA	CCCGATAGACCCATGATGACA
Bc_102	R47	Mut. 19	PTS system trehalose-specific EIIBC component	TCCGATAGCAGGTGCTAATACA	CAACAATCGTTGTGACAGGTG

PCR was performed to amplify and re-sequence specific DNA fragments for selected mutations. The table includes data for each parental strain, evolved line, the gene potentially affected by the mutation, specific oligonucleotide pairs used for amplification.

Supplementary Table S3. Mutations validated by PCR amplification and sequencing

**Parental strain**	**Evolved line**	**Nmutation name**	**Position**	**Change**		**Gen function affected**
Bc_370a	M47	Mut. 1 (A)	Intergenica	G	A	Protein translocase subunit SecA
Bc_102	P47	Mut. 2 (B)	Intergenica	TA	T	Protein SapB
Bc_102	Q47	Mut. 3 (C)	Intergenica	AG	A	DNA-invertase hin
Bs_21	B47	Mut. 4 (D)	Intergenica	GA	A	Citrate synthase 2
Bs_21	B47	Mut. 5 (E)	Intergenica	GT	G	Endoglucanase S
Bs_21	B47	Mut. 6 (F)	Intergenica	CT	C	Spore germination lipase LipC
Bs_90	F47	Mut. 7 (G)	Intergenica	AT	A	hypothetical protein PF09954

Bs_21	B47	Mut. 10 (J)	**Intragenic**	GA	G	DNA replication protein DnaD
Bs_21	B47	Mut. 11 (K)	**Intragenic**	C	CT	RNase_H superfamily (domain)
Bs_90	D47	Mut. 12 (M)	**Intragenic**	G	GA	CdaA regulatory protein CdaR
Bs_90	F47	Mut. 13 (N)	**Intragenic**	G	GTTAACGTTGA	CdaA regulatory protein CdaR
Bc_370a	O47	Mut. 14 (O)	**Intragenic**	A	AG	DNA integrity scanning protein DisA
Bc_102	P47	Mut. 15 (P)	**Intragenic**	G	GC	DNA integrity scanning protein DisA
Bc_111b	T47	Mut. 16 (Q)	**Intragenic**	T	TC	DNA integrity scanning protein DisA
Bc_102	Q47	Mut. 17 (R)	**Intergenic**	A	AT	Protein RecA -
Bs_90	F47	Mut. 18 (S)	**Intragenic**	G	GA	Glycine betaine transport ATP-binding protein OpuAA
Bc_102	R47	Mut. 19 (T)	Intergenic (downstream)	TA	T	PTS system trehalose-specific EIIBC component -

The DNA sequences obtained from PCR amplification were compared to the corresponding segments of the parental strain's genome. In cases where a mutation was present in the evolved strain, alignment of the sequences revealed nucleotide differences. The table includes data for each parental strain, evolved line, the gene potentially affected by the mutation, specific nucleotide changes, and the gene's function potentially impacted.
